# Detection of Heavy Metals, Their Distribution in *Tilapia* spp., and Health Risks Assessment

**DOI:** 10.3390/toxics11030286

**Published:** 2023-03-20

**Authors:** Leonel C. Mendoza, Ronnel C. Nolos, Oliver B. Villaflores, Enya Marie D. Apostol, Delia B. Senoro

**Affiliations:** 1 Resiliency and Sustainable Development Laboratory, Yuchengco Innovation Center, Mapua University, Intramuros, Manila 1002, National Capital Region, Philippines; 2 Food Processing Technology Research and Development Center (FPTRDC), Mindoro State University (MinSU)-Calapan City Campus, Masipit, Calapan City 5200, Oriental Mindoro, Philippines; 3 The Graduate School, University of Santo Tomas, España Blvd, Sampaloc, Manila 1008, National Capital Region, Philippines; 4 College of Teacher Education, Mindoro State University (MinSU)-Calapan City Campus, Masipit, Calapan City 5200, Oriental Mindoro, Philippines; 5 Graduate School, Mindoro State University (MinSU)-Calapan City Campus, Masipit, Calapan City 5200, Oriental Mindoro, Philippines; 6 MIMAROPA Food Innovation Center (FIC), Mindoro State University (MinSU)-Calapan City Campus, Masipit, Calapan City 5200, Oriental Mindoro, Philippines; 7 Mapua-MSC Joint Research Laboratory, Marinduque State College, Boac 4900, Marinduque, Philippines; 8 College of Environmental Studies, Marinduque State College, Boac 4900, Marinduque, Philippines; 9 Research Center for the Natural and Applied Sciences, University of Santo Tomas, Sampaloc, Manila 1008, National Capital Region, Philippines; 10 Department of Biochemistry, Faculty of Pharmacy, University of Santo Tomas, Sampaloc, Manila 1008, National Capital Region, Philippines; 11 College of Business and Management, Mindoro State University (MinSU)-Calapan City Campus, Masipit, Calapan City 5200, Oriental Mindoro, Philippines; 12 School of Civil, Environmental, and Geological Engineering, Mapua University, Intramuros, Manila 1002, National Capital Region, Philippines

**Keywords:** agriculture, fish, health hazards, heavy metals, organs, meat

## Abstract

Concentrations of heavy metals (HMs) were assessed in *Tilapia* spp. from selected communities in Calapan City, Philippines. Eleven (11) inland farmed tilapia samples were collected and analyzed for HMs concentration using X-ray fluorescence (XRF). The 11 fish samples were cut into seven pieces, according to the fish body parts, constituting a total of 77 samples. These fish samples were then labeled as bone, fins, head, meat, skin, and viscera. Results showed that the mean concentration of Cd in all parts of tilapia exceeded the Food and Agriculture Organization/World Health Organization (FAO/WHO) limits. The highest concentration was recorded in the fins, which was sevenfold higher than the limit. The trend of the mean concentration of Cd in different parts of tilapia was fins > viscera > skin > tail > head > meat > bone. The target hazard quotient (*THQ*) recorded a value less than 1. This means that the population exposed to tilapia, within the area where fish samples originated, were not at risk to non-carcinogens. The concentrations of Cu, Pb, Mn, Hg, and Zn in different parts, particularly in skin, fins, and viscera, also exceeded the FAO/WHO limits. The calculated cancer risk (*CR*) in consuming the fish skin, meat, fins, bone, viscera, and head was higher than the USEPA limit. This indicated a possible carcinogenic risk when consumed regularly. Most of the correlations observed between HMs in various parts of the tilapia had positive (direct) relationships, which were attributed to the HM toxicity target organ characteristics. Results of the principal component analysis (PCA) showed that most of the dominating HMs recorded in tilapia were attributable to anthropogenic activities and natural weathering within the watershed of agricultural areas. The agriculture area comprises about 86.83% of the overall land area of Calapan City. The identified carcinogenic risks were associated with Cd. Therefore, regular monitoring of HMs in inland fishes, their habitat, and surface water quality shall be carried out. This information is useful in creating strategies in metals concentration monitoring, health risks reduction program, and relevant guidelines that would reduce the accumulation of HM in fish.

## 1. Introduction

After milkfish, tilapia is the second most farmed fish in the Philippines [1], with an average annual consumption of 4.6 kg per person [2]. The Mozambique tilapia (*Oreochromis mossambicus*), imported from Thailand in 1950, was the first tilapia to be introduced to the Philippines. Meanwhile, the Nile tilapia (*O. niloticus*) was introduced in 1972 and is now the country’s main species of tilapia being farmed [3] (pp. 75–91). In 2019, the tilapia per capita availability for Filipinos was 2.9 kg/y, compared to 2.5 kg/y for milkfish [4] (p. 25). Luzon (mostly freshwater) is the country’s largest producer of farmed tilapia, accounting for 92% of total output (261,210 MT) in the country, followed by Mindanao (freshwater and seawater) at 6%, as well as the Visayas (mostly brackish water) at 2% [1,5]. *Tilapia* aquaculture is one of Calapan City’s inland fishing industries, providing living and a source of food to many residents. The city of Calapan is located on the northeastern coast of the island of Mindoro and the capital city of Oriental Mindoro. It covers an area of 21,730 hectares and is composed of 62 barangays (the smallest administrative local government unit). A total of 18,607.8 hectares (86.83% of the total area) are dedicated to agriculture (rice fields) and fishing, with 1300 hectares (5.98% of the total area) dedicated to inland fishing and the rest to other agricultural, residential, commercial activities, and few industry sectors [6].

Fish, including tilapia, are some of the most consumed animal-derived food items due to their high nutritional content, which includes high-quality animal protein and a variety of minerals and other essential nutrients [7,8,9,10,11]. With food safety being a significant public health concern worldwide, there is a tremendous demand for studies on the safety of food products such as fish consumed by people. Risks connected with consuming of contaminated foods have been a prominent focus of scientific investigation in recent decades [12]. Heavy metals (HMs) are among the most dangerous contaminants in food production and distribution [13,14], including fisheries. These issues are becoming increasingly serious over the world, particularly in developing countries [12].

HMs introduced to the aquatic environment, primarily as a result of human activities [15], accumulate in the water environment and subsequently pass via the food chain to aquatic organisms [16]. The quantities of HMs in fish have been intensively examined in many locations throughout the world during the last several decades [17]. Fish are also employed as bioindicators of aquatic ecosystems to determine the extent of HM pollution and the risk of bioaccumulation to humans [18]. This is because fish are particularly vulnerable to pollution and changes in their surrounding environment [19]. The study of Ali et al. [20] shows that fish, indeed, can be used for the biomonitoring of aquatic ecosystems. 

Several studies have been conducted in the Philippines in recent years to study the level of HM concentrations in selected species of tilapia [21,22,23] and their potential as bioindicators of water pollution [24,25,26]. To the best of the authors’ knowledge, this is the first study that investigated the level of concentrations of HMs in meat and various organs of *Tilapia* spp. in Calapan City and even in the whole province of Oriental Mindoro. With that, this study aimed to (i) provide baseline data on the distribution of HMs in various parts of *Tilapia* spp. samples from selected communities in Calapan City, Philippines and (ii) assess the health risks associated with the consumption of the investigated tilapia samples. The levels of HMs in the fish samples can give preliminary insights on the level of aquatic pollution in the area, while the result of the health risks assessment will have implications on food safety and quality.

## 2. Materials and Methods

### 2.1. Study Area and Sample Collection

**Figure 1:**
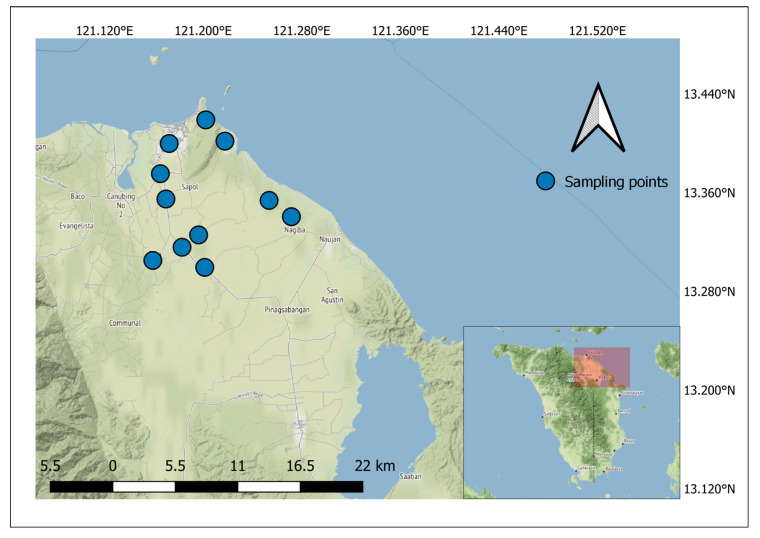
Map of the study area and location of sampling sites.

Eleven (11) samples of *Tilapia* spp. were collected from vendors in the barangays of Calapan City, as described by the global positioning system (GPS) plots in Figure 1. These are the barangays that had available *Tilapia* spp. that could spatially represent Calapan City. This city is about 73.28 nautical miles (about 5 h of travel by land and sea) from Manila, the Philippines’ capital city. Barangays are the most basic territorial, administrative, and local government units in the Philippines [21]. In addition, GPS coordinates for collection sites were recorded to map the source or origin of fish samples. The eleven (11) barangays from which the fish samples were collected were: Bayanan II, Biga, Comunal, Lalud, Managpi, Nag-iba I, Nag-iba II, Panggalaan, Parang, Sta. Isabel, and Suqui.

### 2.2. Sample Collection, Preparation and Analysis

**Figure 2:**
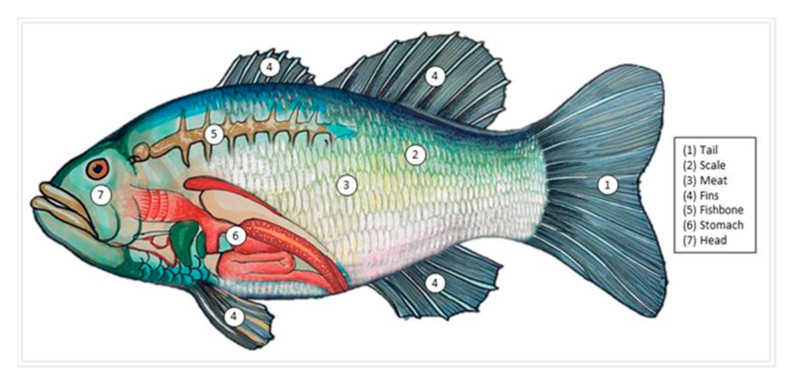
Different parts of *Tilapia* spp. Collected for HM analysis.

Approximately one (1) kilogram of each *Tilapia* spp. of the same size (length of ±7”) from eleven (11) sampling sites were bought from local fish vendors. The collected samples were wrapped in a cleaned plastic container, sealed, labeled, and kept in an icebox cooler before transporting them to MIMAROPA Food Innovation Center. The fish samples, bought from 11 sites, were cut into seven parts, namely, the tail, scale/skin, meat, fins, bones, viscera/stomach, and head, and they were weighed, grouped, and labeled according to fish parts, such as bone, fins, head, meat, skin, tail, and stomach/viscera. The 11 *Tilapia* spp. fish samples became 77 samples for metal analysis. This is to identify which part of the *Tilapia* spp. contains the lowest and the highest metals concentration. Figure 2 illustrates the tilapia fish and its various parts. The 77 samples were then dried in the Electric Dehydrating Memmert UF750 oven with the aid of a tray. The oven was set at 65 °C, and the fish samples were dried for eight hours. After drying, the tilapia fish samples were powdered using the Dowell Portable Blender. Powdered samples were then placed in resealable plastic bag No. 2 (60 × 85 × 0.04 mm) and labeled accordingly. The 77 powdered samples, with mean dry weight shown in Table 1, were prepared following the USEPA 823-B-00-007 [27]. The typical minimum sample weight for Vanta XRF analysis is 5 g. The sample weight does not affect the elemental detection capability and sensitivity of XRF. The fish samples were analyzed for their metals concentrations using the portable Olympus Vanta X-ray Fluorescence (XRF) spectrometer, of the Yuchengco Innovation Center of Mapua University, at the Food Processing Technology Research and Development Center of Mindoro State University Calapan City Campus. The XRF, set to soil mode, was calibrated with the aid of the manufacturer using the Olympus Vanta XRF standard reference in a No. 2 resealable plastic bag, together with the ICP-OES results of comparable set of samples. ICP-OES has been internationally accepted as the primary instrument for elemental analysis. Hence, this study used ICP-OES as a calibration aid to ensure an accurate result of the XRF. The new model, as a result of calibration to adjust default calibration values and meet site specific factors, has been labelled as ‘soil ziploc mode’. After this, the portable XRF is ready for elemental analysis and becomes a reliable instrument for metal detection in this study for various media, and it has been used in various studies [28,29,30,31,32,33,34]. The 77 samples were analyzed for the presence of ten (10) metals, such as arsenic (As), barium (Ba), cadmium (Cd), copper (Cu), iron (Fe), lead (Pb), manganese (Mn), mercury (Hg), nickel (Ni), and zinc (Zn). The limit of detection (LOD) of XRF with respect to the identified HMs is shown in Table 2. All extracted data values showing “LOD” were represented by a number that is one step lower than the XRF LOD value shown in Table 2. This is to represent numeral values in calculating the quantitative health risks.

### 2.3. Health Risk Assessment

The health risk was assessed using the chronic daily intake (*CDI*), target hazard quotient (*THQ*), and cancer risk (*CR*). These were calculated to estimate the various health consequences associated with HM consumption of tilapia, particularly its meat.

#### 2.3.1. Chronic Daily Intake (EDI)

The *CDI* of all HMs detected through ingestion of various parts of tilapia was determined following Equation (1) [28].

(1) $$CDI=\frac{C_{m}\times IR\times EF\times ED}{BW\times AT}$$

where *C_m_* is the concentration of HMs (mg/kg), *IR* is the ingestion rate of various parts (kg/person/day) (Table 3), *EF* is the exposure frequency (365 days/year) [28], *ED* is exposure duration (70 years) [28], *BW* is the average body weight of an adult Filipino (70 kg) [28], and *AT* is the averaging time (*EF* × *ED*). 

#### 2.3.2. Target Hazard Quotient (*THQ*)

The non-carcinogenic health risk (NCHR) posed by the consumption of the various parts of tilapia contaminated with HMs was defined by *THQ* and was estimated by following Equation (2) [28,38].

(2) $$THQ=\frac{CDI}{R_{f}D}$$

where *R_f_D* is the reference dose of metals that provides an estimate of the daily exposure of an individual to a particular contaminant such as HM with a potential non-carcinogenic health risk [39] The *R_f_Ds* for As, Ba, Cd, Cu, Fe, Pb, Mn, Hg, Ni, and Zn are 0.0003, 0.2, 0.001, 0.04, 0.7, 0.0035, 0.14, 0.0001, 0.02, and 0.3 mg kg^−1^, respectively [40,41,42,43,44,45,46]. Generally, a *THQ* < 1 implies very little to no non-carcinogenic health effects. This means no adverse health effect is expected for the population. A *THQ* of ≥1 means that the population exposed to the metals hazards in fish is at risk. 

To understand the overall non-carcinogenic health risks due to the intake of various parts of tilapia, the total target hazard quotient (*TTHQ*) was calculated following Equation (3) [21].

*TTHQ* = *THQ_As_* + *THQ_Ba_* + *THQ_Cd_* + *THQ_Cu_* + *THQ_Fe_* + *THQ_Pb_* + *THQ_Mn_* + *THQ_Hg_* + *THQ_Ni_* + *THQ_Zn_* (3)

As with the *THQ*, a computed *TTHQ* of <1 implies no adverse health effect is expected. A *TTHQ* of ≥1 implies that the exposed population is at risk.

#### 2.3.3. Cancer Risk (*CR*)

To assess the potential carcinogenic risk due to lifetime exposure to carcinogens, Equation (4) was followed [47].

(4) CR=CDI×SF

where *SF* (mg kg^−1^ day^−1^) denotes the cancer slope factors set by the United States Environmental Protection Agency(USEPA) for a certain carcinogen. In this study, only As, Cd, Pb, and Ni were included in the calculation of *CR*, as they were identified as carcinogens by the USEPA [48]. The *SF* values for As, Cd, Pb, and Ni are 1.5, 6.3, 0.0085, and 0.84 mg kg^−1^ day^−1^, respectively [28,49,50]. *CR* values greater than 1 × 10^−4^ indicate that there is a high probability of the occurrence of cancer risk in the population [51].

### 2.4. Data Analysis

Descriptive statistics of the HM concentration in various parts of tilapia were provided using Microsoft Excel 2016. The concentrations of HMs in tilapia were Box-Cox transformed prior to multivariate analysis [52] using IBM SPSS Statistics 23 to ensure that the data were normally distributed [53]. One-way ANOVA was employed to indicate the significant differences in HMs in various parts of tilapia. To understand the relationships of HMs in various parts of tilapia, a correlation matrix (CM) in the form of a correlogram was performed using RStudio. Similarly, principal component analysis (PCA) was employed using PAST 4.03 to further support the result of the CM and evaluate the possible sources of HMs in the study area. The principal components (PC), which had eigenvalues greater than 1 and comprised >70% of the total variance, were retained after the PCA [53,54].

## 3. Results

### 3.1. Concentration of HMs in Different Parts of the Fish

X-ray fluorescence (XRF) analysis was used to determine the levels of HMs (As, Ba, Cd, Cu, Fe, Pb, Mn, Hg, Ni, and Zn) in tilapia collected from eleven (11) barangays in Calapan City, Oriental Mindoro, Philippines. The mean concentrations of these HMs in the tail, skin, meat, fins, bone, stomach, and head of the tilapia are shown in Table 4. 

**Figure 3:**
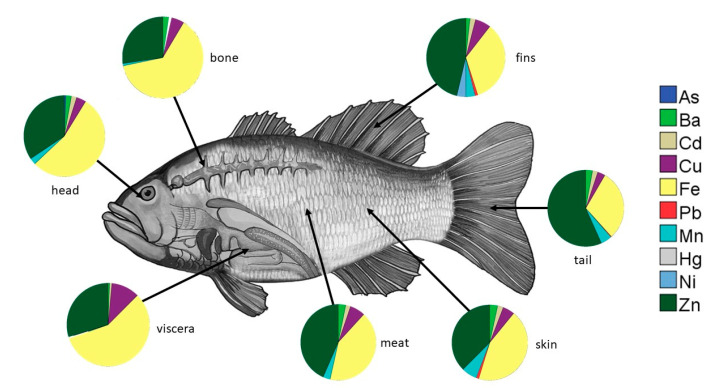
The share of HM concentration in each part of *Tilapia* spp.

The mean concentration of Cd in all parts of tilapia exceeded the FAO/WHO limit [55,56]. The highest concentration was recorded in the fins, which was sevenfold higher than the limit. The mean concentration of Cd in different parts of tilapia is in decreasing order of fins > viscera > skin > tail > head > meat > bone. For the Cu concentration, only the concentration in the viscera exceeded the FAO/WHO limit [55,56]. The mean concentration of Cu in several parts of tilapia is in the following order: viscera > fins > skin > meat > bone > tail > head. Similarly, the mean concentrations of Pb in the skin and fins of tilapia exceeded the FAO/WHO limit [55,56]. The mean concentrations of Mn in all parts of tilapia except for the bone exceeded the limit of 1. The Mn concentration in the parts of tilapia follows the order of skin > fins > tail > meat > head > viscera > bone. For the mean concentration of Hg, only the concentration in viscera exceeded the FAO/WHO limit [55,56]. On the other hand, only the mean concentration of Zn in the viscera of tilapia exceeded the allowable limit. Results of the one-way ANOVA (Table 5) show that the accumulation of Ba, Cu, Mn, and Zn in various parts of the tilapia was significantly different (*p* < 0.005). Figure 3 shows the concentration (%) of HMs in each part of the tilapia. Fe and Zn were recorded to have the highest concentration in each part.

### 3.2. Health Risk Assessment

#### 3.2.1. Non-Carcinogenic Health Risk (NCHR) Assessment

**Figure 4:**
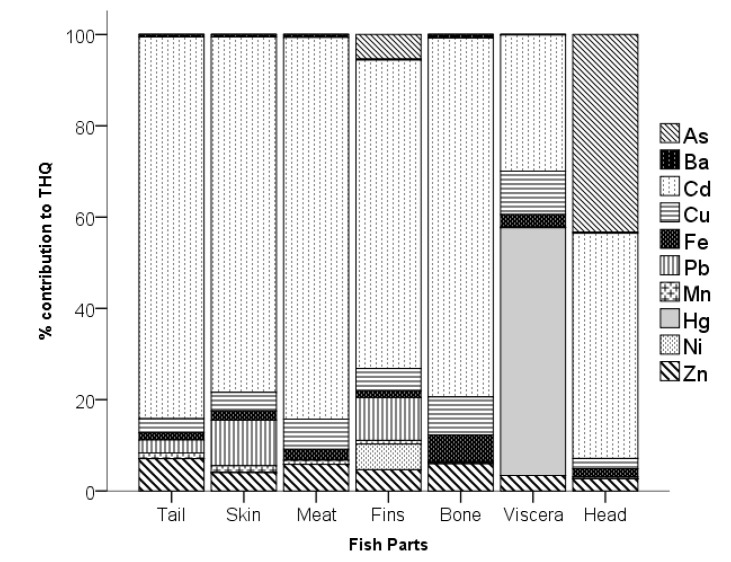
Percent (%) contribution of HMs to the *THQ* per body part.

The NCHR of HMs to the locals of Calapan City is shown in Table 6. The NCHR of the HMs in the various parts of tilapia, as represented by the target hazard quotient (*THQ*), did not exceed 1. This means that the population exposed to the fish is not at risk [21] to non-carcinogens. The highest *THQs* were recorded for Hg in the viscera at 0.0822 and Cd in the meat at 0.0805. As shown in Figure 4, the highest percent (%) contribution to the *THQ* of the tail, skin, meat, fins, bone, and head was due to Cd, which ranged from 49.34–83.63%. On the other hand, Hg contributed much to the *THQ* of the viscera at 54.26%. Generally, the *THQ* of HMs in various parts of tilapia follows the decreasing order of Cd > Hg > As > Cu > Zn > Pb > Fe > Ni > Mn > Ba. 

#### 3.2.2. Carcinogenic Risk Assessment

**Figure 5:**
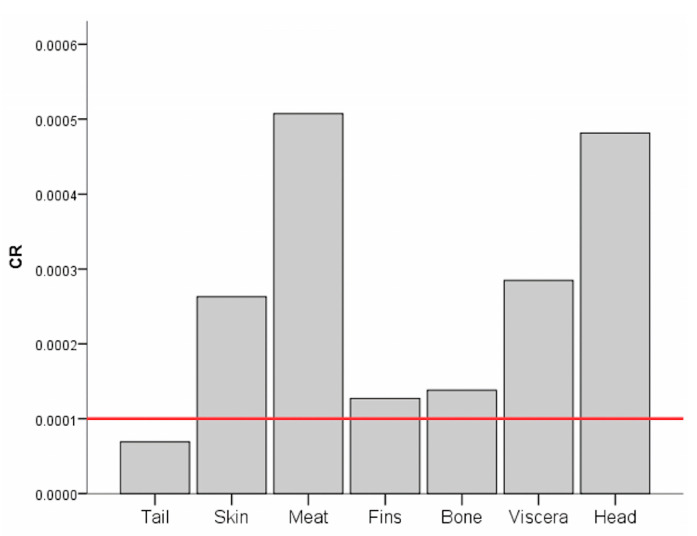
*CR* of HMs in various parts of *Tilapia* spp.

The carcinogenic risk (*CR*) of consuming various parts of tilapia contaminated with As, Cd, Pb, and Ni was assessed. As shown in Figure 5, except for the tail, all of the *CR* through the consumption of several parts of tilapia exceeded the threshold value of 1 × 10^−4^, implying that cancer risk was highly probable [51]. The highest *CR* was recorded in the meat, which was approximately 0.00051. This was followed by the head at 0.00048. The sequence of *CR* in various parts of tilapia followed the order of meat > head > viscera > skin > bone > fins > tail. 

**Figure 6:**
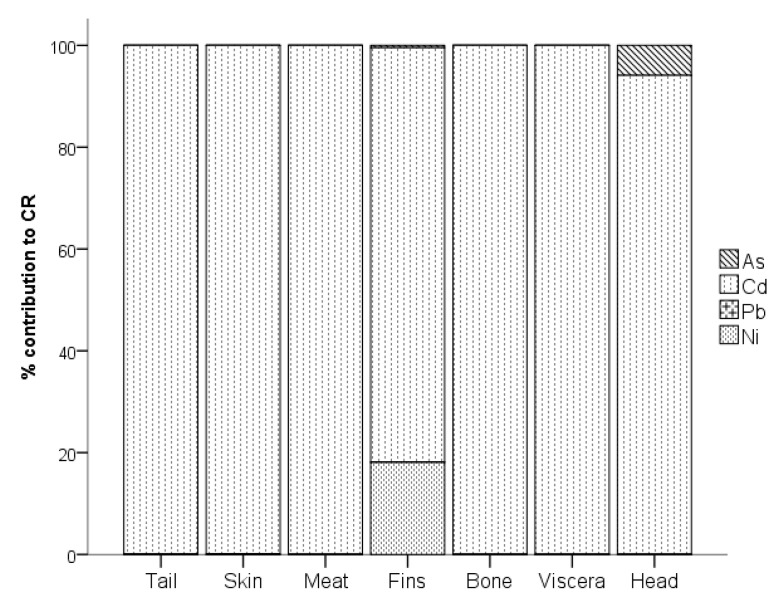
Percent (%) contribution of As, Cd, Pb, and Ni to CR.

Figure 6 shows the % contribution of each HM identified as a carcinogen to the *CR*. More than 99% of the *CR* in the tail was contributed by Cd. Similarly, the Cd contributed 99.94%, 100%, 81.40%, 100%, 100%, and 94.10% to the *CR* in the skin, meat, fins, bone, viscera, and head, respectively, of tilapia. It also contributed 5.90% to the *CR* in the head, while Ni contributed 18.09% to the *CR* in the fins. 

### 3.3. Relationship of HMs in Various Parts of Tilapia *spp.*

**Figure 7:**
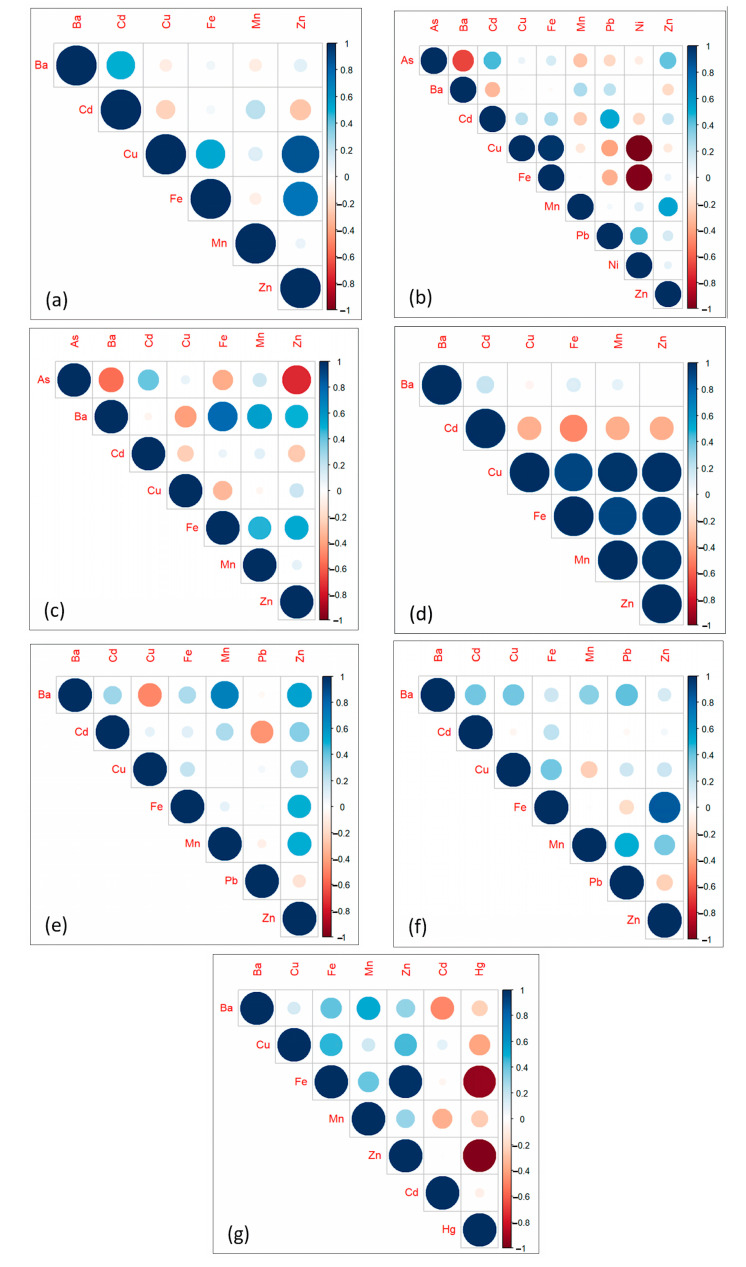
Correlogram of HMs in the (**a**) bone, (**b**) fins, (**c**) head, (**d**) meat, (**e**) skin, (**f**) tail, and (**g**) viscera of *Tilapia* spp.

Figure 7 shows the correlation of HMs in different parts of tilapia. As shown in Figure 7a, a high positive correlation exists between Cu-Zn (0.865, 0.01 level) and Fe-Zn (0.732, 0.05 level) in the bone of tilapia. In the fins, a moderate negative correlation was observed between As-Ba (−0.663, 0.05 level), while a very high negative correlation was observed between Cu-Ni (−0.998, 0.01 level) and Fe-Ni (−0.976, 0.01 level). On the other hand, there is a very high positive correlation between Cu-Fe (0.972, 0.01 level) (Figure 7b). In Figure 7c, high positive correlation was observed between Ba-Fe (0.780, 0.01 level), while a high negative correlation was observed between As-Zn (−0.740, 0.01 level) in the head of the tilapia. Similarly, there are very high positive correlations between Cu-Fe (0.916), Cu-Mn (0.975), Fe-Mn (0.916) Fe-Zn (0.952), and Mn-Zn (0.979) in the meat of tilapia, which are significant at 0.01 level (Figure 7d). In Figure 7e, a moderate positive correlation was observed between Ba-Mn (0.680) in the skin, which is significant at the 0.05 level, while a high positive correlation was observed between Fe-Zn (0.842, 0.01 level) in the tail (Figure 7f). In Figure 7g, three (3) significant correlations were observed in the viscera of tilapia—a very high negative correlation between Fe-Hg (−0.939) and Hg-Zn (−0.976) and a very high positive correlation between Fe-Zn (0.987). All the observed correlations were significant at the 0.01 level.

**Figure 8:**
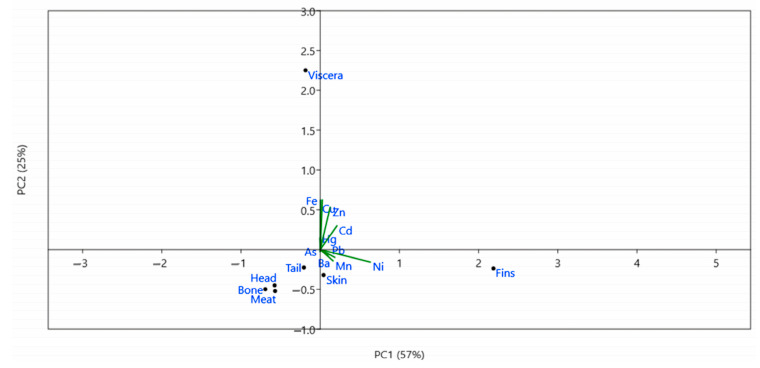
PCA biplot of HMs in various parts of *Tilapia* spp.

Furthermore, PCA was carried out to evaluate if the sources of HM contamination in tilapia are either due to anthropogenic or natural causes [57]. Principal component (PC) 1 explained 57.23% of the total variance and exhibited an eigenvalue of 7.13 (Figure 8). Ni dominated PC1 with a loading of 0.8684. Similarly, PC2 explained 24.81% of the total variance with an eigenvalue of 3.09. Cu and Fe dominated PC2 with loadings of 0.56193 and 0.5651, respectively. PC3, on the other hand, has an eigenvalue of 2.05 and explained 16.46% of the total variance. PC3 was dominated by Ba and Mn with loadings of 0.6399 and 0.5392, respectively. 

## 4. Discussion

Several parts of tilapia in Calapan City, Philippines were analyzed for the concentration of heavy metals (HMs). The Cd concentration in all parts of tilapia exceeded the FAO/WHO limit, indicating possible health risks to consumers. Cd is a highly toxic HM that can adversely affect organisms even at low concentrations [58]. Generally, Cd is ubiquitous in the environment, but its concentration increases due to anthropogenic causes, such as fertilizer application, sewage, batteries, and pigments [59,60]. Elevated concentrations of Cd in the meat and gills of *Oreochromis niloticus* were also observed in Southwestern Nigeria, attributed to the contaminated river where the fish was caught [61]. Some organ systems mainly targeted by Cd in humans are the urinary, skeletal, and respiratory systems, which may result in renal tubular dysfunction and osteomalacia when continuously exposed to high concentrations [62]. Moreover, a high concentration of Mn was also observed in all the parts of the tilapia except in the bone. Similar to Cd, Mn is also ubiquitous in the environment, and it originates from a variety of sources, including agricultural, industrial, and urban pollution [63]. The study area is mainly an agricultural land, and the high concentrations of Cd and Mn can be attributed to this condition. The highest concentrations of Cu, Hg, and Zn were found at the viscera of tilapia. Aquatic organisms such as fish are vulnerable to the bioaccumulation of metals present in the water. Hence, the elevated concentrations of metals in fish samples were attributable to the fish pond’s water quality. It is estimated that most of the total Hg in the meat and organs of fish such as tilapia is methylmercury (MeHg), which is the most toxic form of Hg. Th MeHg usually binds with the cysteine-rich tissues of the fish, particularly the meat and viscera [64], such as the recorded data of the fish samples in this study. However, a study focusing on speciation is helpful in identifying the species of metals found in tilapia species. Cu and Zn, on the other hand, are considered essential metals, but they can pose adverse health effects to humans in excessive amounts [65]. This is comparable to the results of other studies in an island province in the Philippines [21], Hong Kong [66], and Malaysia [67]. The common site of metal accumulation in fish is the viscera where food is stored and processed. Additionally, metal metabolism in the fish played a role in this condition [68]. Additionally, the HMs ingested by fish are stored in the kidney and liver, and some excessive metals remain in the fish’s viscera [66]. 

In addition, it was observed that external parts of the tilapia, such as the tail, scale, and fins, recorded as accumulated, and they had the highest mean concentration of all the detected HMs. This finding may be implied by the fact that these parts are in direct contact with water, from which the fish absorb HMs [69]. Meanwhile, on average, meat recorded minimal absorption of HMs compared to tail, scale, fins, and viscera. This is consistent with the findings of Ju et al. [70] and Abdel-Baki et al. [71]. This is because fish meat is considered a passive location of metal biotransformation and build-up [17,72].

Meanwhile, none of the HMs in the various parts of tilapia exceeded the *THQ* of 1 [21]. Ishak et al. (2020) [60] also determined the concentration of Pb and Cd in tilapia (*Oreochromis niloticus*) from Kuala Lumpur, Malaysia and found that the *THQs* for these HMs were less than 1. However, the highest *THQs* were recorded for Hg in the viscera and Cd in the meat. The high *CDIs* and low reference doses of Hg and Cd contributed to the high *THQs* [40]. A high concentration of Hg was also observed in the viscera of clupeid (*Brevoortia tyrannus*) caught in the northeast bay of the United States of America. Hence, evisceration of the fish prior to cooking or processing was recommended by the authors [73]. To prevent the possible non-carcinogenic health risk due to the intake of Cd in the meat of tilapia, the authors recommend that consumption of this part should not exceed 0.048 kg/person/day. Results of the *CR*, on the other hand, showed that there is potential cancer risk via ingestion of the fish’s skin, meat, fins, bone, viscera, and head. The high *CR* in these parts was primarily attributed to the high concentration and slope factor of Cd [50]. Chronic and acute exposures of humans to high concentration of Cd can potentially cause lung, kidney, and pancreatic cancers [74]. 

The result of the correlogram in each part of the tilapia showed that most of the HMs analyzed had a significant positive correlation. This implies that, when a particular HM tends to increase, the other correlated variable (i.e., HM) does the same. This is also an indication that the correlated HMs may have a similar source of contamination [75]. Similarly, the result of the PCA showed that there were three (3) major PCs observed. PC1 was dominated by Ni. Generally, food is the major source of human exposure to Ni. Common anthropogenic sources of Ni to the environment include dust or fumes from power plants, waste incinerators, welding industries, and pesticides [76,77]. Through deposition, this Ni may be transported to aquatic environments such as rivers and lakes, where fish and other aquatic organisms may be exposed. PC2, on the other hand, was dominated by Cu and Fe. These HMs are naturally abundant due to the weathering of rocks. However, Cu concentration in the environment, particularly in water systems, increases due to major anthropogenic sources such as mining, agriculture, waste treatment plants, and industrial and municipal solid waste [78]. Furthermore, the dominant HMs in PC3 were Ba and Mn. Both Ba and Mn are also naturally occurring in the environment from weathered rocks, primarily sedimentary rocks. However, their concentration in the environment increases due to various man-made sources such as paints, oils, pesticides/fertilizers, manufacturing, and mining [79,80]. Overall, the PCA revealed that HM contamination in the investigated tilapia samples can be attributed to anthropogenic causes, especially agricultural run-offs. To emphasize, the tilapia collected from Panggalaan and Bayanan II recorded the highest total HM content. These sampling sites are comprised of numerous agricultural fields, mills, resorts, and other industrial establishments. Elevated concentrations of HMs were also recorded in fish collected in rivers that receive run-off from agricultural fields, such as in Bangladesh [81], Iran [82], and Mozambique [83].

It is also important to note that tilapia is commonly cultured or farmed in fishponds. The source of the water and the feeds given to the fish probably contribute to the concentration of HMs in various parts of tilapia. Yilmaz et al. [84] and Al-Majed et al. [85] emphasized that fish habitat and feeding habits may influence these varying levels of HM concentration. Hence, it is highly recommended to regularly monitor the water quality of these fishponds to mitigate the bioaccumulation of HMs in tilapia and reduce or eliminate the possibility of human health risks. Additionally, physiological conditions, types of tissues analyzed, growing rate, age, gender, and size were also contributing factors for the varying HM concentrations in fish [65,86,87].

## 5. Conclusions

This study investigated the levels of heavy metals (HMs) in various parts of *Tilapia* spp. in Calapan City, Province of Oriental Mindoro, Philippines. The mean concentrations of Cd and Mn in all parts of the tilapia exceeded the FAO/WHO limits. Similarly, the mean concentrations of Cu, Fe, and Hg in the viscera exceeded the limits recommended by FAO and WHO. An elevated concentration of Pb was recorded in the skin and fins of tilapia. The result of the calculated *CR* recorded that consumption of tilapia, specific to the fish’s skin, meat, fins, bone, viscera, and head, may pose a potential cancer risk, as the *CR* of these fish parts exceeded the threshold value of 1 × 10^−4^ set by USEPA. The correlogram showed that most of the correlations between HMs in various parts of the tilapia had a positive (direct) relationship, which is attributed to the HMs toxicity target organ characteristic. Regular monitoring of HM concentration in tilapia, the inland fish habitat, and surface water quality shall be carried out. 

## Figures and Tables

**Table 1:** The mean dry weight of fish parts, *n* = 77.

Fish Part	Mean Dry Weight (g)
Bone	74.73
Fin	9.73
Head	54.71
Meat	40–92
Skin	69.64
Tail	6.27
Viscera	5.00

**Table 2:** Limit of detection (LOD) of Olympus Vanta XRF [35].

Name of Metals	LOD (mg/kg)
As	1
Ba	15
Cd	2
Cu	1
Fe	1
Hg	1
Mn	3
Ni	3
Pb	1
Zn	1

**Table 3:** Estimated ingestion rate of various parts of *Tilapia* spp. [36,37].

Part	Ingestion Rate (kg Person^−1^ Day^−1^)
Bone	1.53 × 10^−3^
Fin	3.01 × 10^−4^
Head	2.91 × 10^−3^
Meat	3.88 × 10^−3^
Skin	9.74 × 10^−4^
Tail	3.01 × 10^−4^
Viscera	9.59 × 10^−3^

**Table 4:** Mean concentration of HMs (mg kg^−1^) in various parts of *Tilapia* spp.

Part	As	Ba	Cd	Cu	Fe	Pb	Mn	Hg	Ni	Zn
Tail	ND	3.27 ± 1.2	2.55 ± 2.0	3.73 ± 3.5	34.00 ± 29.1	0.30 ± 0.5	5.39 ± 9.5	ND	ND	64.56 ± 55.3
Skin	ND	4.14 ± 0.8	3.00 ± 2.2	6.28 ± 5.4	54.04 ± 47.9	1.35 ± 1.0	8.22 ± 11.7	ND	ND	46.28 ± 32.3
Meat	ND	2.23 ± 0.5	1.45 ± 0.9	4.61 ± 7.5	29.17 ± 30.1	ND	2.17 ± 4.82	ND	ND	30.38 ± 52.8
Fins	0.09 ± 3	2.90 ± 0.7	3.82 ± 5.8	11.02 ± 38.2	58.67 ± 124.2	1.85 ± 5.4	6.43 ± 6.4	ND	6.36 ± 2.11 ND	78.65 ± 57.7
Bone	ND	1.91 ± 0.7	1.00 ± 1.3	4.22 ± 3.6	52.48 ± 41.9	ND	0.74 ± 0.14	ND	ND	22.73 ± 14.8
Viscera	ND	2.25 ± 0.7	3.30 ± 1.9	41.95 ± 73.3	216.50 ± 566.9	ND	1.77 ± 5.1	0.60 ± 1.9	ND	112.53 ± 332
Head	0.45 ± 5	1.80 ± 0.6	1.73 ± 1.9	3.13 ± 3.1	44.00 ± 59.6	ND	2.09 ± 3.1	ND	ND	27.92 ± 18.8
FAO/WHO Limits [55,56]	1.4	N/A	0.5	30.0	100.0	0.5	1.00	0.50	30.0	100.0

ND—Not Detected.

**Table 5:** One-way ANOVA of the HMs in various parts of *Tilapia* spp.

HM	df	F	*p* Value
Ba	6	11.47	6.81 × 10^−9^
Cd	6	1.456	0.2062
Cu	6	3.781	0.0026
Fe	6	1.837	0.1043
Mn	6	4.234	0.0011
Zn	6	5.151	0.0002

**Table 6:** *THQ* of HMs in each part of *Tilapia* spp.

Parts	As	Ba	Cd	Cu	Fe	Pb	Mn	Hg	Ni	Zn
Tail	0	0.0001	0.0110	0.0004	0.0002	0.0004	0.0002	0	0	0.0009
Skin	0	0.0003	0.0417	0.0022	0.0011	0.0053	0.0008	0	0	0.0021
Meat	0	0.0006	0.0805	0.0064	0.0023	0.0000	0.0009	0	0	0.0056
Fins	0.0013	0.0001	0.0164	0.0012	0.0004	0.0023	0.0002	0	0.0014	0.0011
Bone	0	0.0002	0.0219	0.0023	0.0016	0	0.0001	0	0	0.0017
Viscera	0	0.0002	0.0452	0.0144	0.0042	0	0.0002	0.0822	0	0.0051
Head	0.0631	0.0004	0.0719	0.0033	0.0026	0	0.0006	0	0	0.0039

## Data Availability

All data are contained in the manuscript.
